# Prognostic value of microRNA expression levels in pancreatic adenocarcinoma: a review of the literature

**DOI:** 10.18632/oncotarget.20277

**Published:** 2017-08-16

**Authors:** Patrick Wald, X. Shawn Liu, Cory Pettit, Mary Dillhoff, Andrei Manilchuk, Carl Schmidt, Evan Wuthrick, Wei Chen, Terence M. Williams

**Affiliations:** ^1^ The Ohio State University Medical Center, Arthur G. James Comprehensive Cancer Center and Richard J. Solove Research Institute, Columbus, OH 43210

**Keywords:** pancreatic cancer, miRNA, non-coding RNA, chemotherapy, prognostic biomarker

## Abstract

**Background:**

Clinical and pathologic markers of prognosis and patterns of failure help guide clinicians in selecting patients for adjuvant therapy after surgical resection for pancreatic adenocarcinoma (PDAC). Recent studies have reported the prognostic utility of microRNA profiling in numerous malignancies. Here, we review and summarize the current literature regarding associations between microRNA expression and overall survival in PDAC patients.

**Materials and Methods:**

We conducted a systematic search in the PubMed database to identify all primary research studies reporting prognostic associations between tumor and/or serum microRNA expression and overall survival in PDAC patients. Eligible articles were reviewed by the authors and relevant findings are summarized below.

**Results:**

We found 53 publications that fit our search criteria. In total, 23 up-regulated and 49 down-regulated miRNAs have been associated with worse overall survival. MiR-21 is the most commonly reported miRNA, appearing in 19 publications, all of which report aberrant over-expression and association with shorter survival in PDAC. Other miRNAs that appear in multiple publications include miR-10b, −21, −34a, −155, −196a, −198, −200c, −203, −210, −218, −222, and −328. We summarize the preclinical and clinical data implicating these miRNAs in various molecular signaling pathways and cellular functions.

**Conclusions:**

There is growing evidence that miRNA expression profiles have the potential to provide tumor-specific prognostic information to assist clinicians in more appropriately selecting patients for adjuvant therapy. These molecules are often aberrantly expressed and exhibit oncogenic and/or tumor suppressor functions in PDAC. Additional efforts to develop prognostic and predictive molecular signatures, and further elucidate miRNA mechanisms of action, are warranted.

## INTRODUCTION

Pancreatic adenocarcinoma (PDAC) is the ninth most common human malignancy and fourth leading cause of cancer-related death in the United States, accounting for approximately 46,000 new diagnoses and 40,000 deaths annually [1]. Deaths are increasing, and by 2030, PDAC is estimated to be the second leading cause of cancer-related death [2]. Approximately 15–20% of pancreatic cancer patients present early enough to be considered candidates for gross surgical resection [3]. For surgically resectable patients, the current standard of care is surgery followed by adjuvant gemcitabine-based chemotherapy, a strategy that is supported by the results of a large randomized trial (CONKO-001) which demonstrated improved overall survival (OS) associated with the use of gemcitabine after complete tumor resection [4]. More recently, results of the ESPAC-4 trial demonstrate that the addition of 5-fluorouracil to gemcitabine results in further improvements in survival [5].

Unfortunately, even after radical surgery, prognosis is very poor, with median OS of 17–21 months and five year OS of approximately 20%. Local-regional recurrence (LRR) rates after surgery remain high, ranging from 23% to 63% [6]. While there is an established benefit to adjuvant chemotherapy, high rates of distant recurrence have called into question the importance of additional local-regional control with regard to improving overall survival. Indeed, prior trials did not clearly demonstrate a benefit with regard to post-operative chemoradiotherapy (CRT), and thus adjuvant CRT is not routinely recommended for localized pancreatic cancer at many centers [7, 8]. The 2016 ASCO consensus guidelines recommend consideration of post-operative radiotherapy in cases with microscopic residual disease and pathologic nodal involvement [9]. Taken together, the issue of adjuvant chemoradiation after resection of pancreas cancer remains controversial.

Recent efforts have focused on clinical and pathologic features that predict patterns of failure and prognosis after surgery, in hopes that improved patient selection for adjuvant therapy may improve outcomes and/or spare toxicity from therapies which do not provide benefit [10]. In addition to clinical and pathologic predictors, there has been a growing emphasis on molecular profiling of pancreatic cancer. Molecular profiling techniques have identified oncogenic and tumor suppressor genes that influence cancer development, patterns of spread, therapeutic response, and prognosis. While many molecular techniques have focused on DNA, microRNA (miRNA) has emerged as a new class of biomarkers which exhibits various oncogenic and tumor suppressor functions in a wide variety of human cancers. These small, non-coding RNA molecules function via messenger RNA (mRNA) silencing and post-transcriptional gene regulation. When bound to a complementary mRNA sequence in the cytoplasm, miRNA promotes mRNA degradation and subsequent suppression of protein translation. MicroRNA dysregulation is a well-documented factor involved in carcinogenesis, with loss of normal function resulting in altered expression of important oncogenes and tumor suppressor genes [11]. In addition to being identified in tumor tissue, miRNAs are stable and readily detectable in the blood, providing the potential for a non-invasive biomarker test.

Aberrant miRNA expression has been implicated in various disease states, including multiple human malignancies [12]. Each unique miRNA has multiple potential mRNA targets, and therefore can have complex effects on malignant cells [13]. As we learn more about miRNA expression levels and downstream signaling effects, miRNA may have the potential to detect malignancy, predict patterns of spread, and provide valuable prognostic information. With regard to pancreatic cancer, miRNA expression profiles have the potential to allow us to better predict local-regional predominant disease that may derive the most benefit from additional local-regional therapy. Conversely, it could select which patients are more likely to recur distantly and, therefore, unlikely to benefit from additional local-regional therapy. Additionally, an improved understanding of miRNA expression in pancreatic cancer can potentially help identify novel molecular targets. Here, we review the current body of literature concerning miRNAs as prognostic biomarkers with regard to overall survival in pancreatic cancer. We will summarize clinical and pre-clinical data that implicate specific miRNAs in various molecular and cellular functions.

## MATERIALS AND METHODS

A systematic search in the PubMed database was performed to identify primary research studies published before November 2016 using the following search terms: (((MicroRNAs OR miRNAs OR Micro RNA OR MicroRNA OR Micro RNAs OR miRNA)) AND (PDAC OR pancreatic ductal adenocarcinoma OR pancreatic cancer OR pancreatic neoplasms OR (pancreas AND cancer) OR (pancreas AND neoplasms))) AND (surgery OR surgical procedure OR operative procedure OR operative surgical procedures OR pancreatectomy OR resection). We included studies that fulfilled the following criteria: (1) studied miRNA expression levels in pancreatic cancer tissue samples, (2) studied miRNA expression levels as a predictive and/or prognostic factor in patients with pancreatic cancer; (3) study was conducted using human samples, tissue or serum. The results of the initial search were screened, based on their title and abstracts, to find publications that fit the inclusion criteria. The full texts of the articles of interest were then evaluated. Those which fit our inclusion criteria and were available in English were considered for the review. Abstract-only articles and meta-analyses were included in our review, and were noted as such.

### Summary of studies

We identified a total of 53 studies fulfilling our search criteria. The sections below summarize the individual miRNAs identified in these studies as having potential oncogenic (Table 1) or tumor suppressive (Table 2) roles. In addition, we have summarized studies that have reported multi-miRNA prognostic molecular signatures (Table 3). Finally, we provide a discussion of the more frequently reported miRNAs, their proposed targets, and their potential molecular and cellular functions.

**Table 1:** List of up-regulated (potentially oncogenic) microRNAs in pancreatic cancer

miRNA	First author	Proposed molecular target(s)	Proposed mechanism(s) and comments
miR-10b	Nakata		
	Preis		
	Frampton		
	miRTarBase	*HOXD10, KLF4, PPARA, NF1*	
miR-17	Yu		Promotes cell proliferation and invasion
	miRTarBase	*PTEN, BCL2L11, CDKN1A*	
miR-21	Hu		
	Dillhoff		
	Kadera		Promotes cell invasion
	Nagao	*PDCD4, TIMP3*	
	Giovannetti	*PTEN, MMP-2, MMP-9, VEGF*	Promotes cell invasion and reduces apoptosis via Akt phosphorylation
	Dhayat	*PTEN, MDR-1*	
	Hwang		
	Jamieson	*Bcl-2, PTEN, Mapsin*	Correlation only seen in the setting of adjuvant chemotherapy
	Frampton	*PDCD4, BTG2, NEDD4L*	
	Liu		
	Wang	*FasL*	
	Ma		Inhibits gemcitabine-induced apoptosis
	Papaconstantinou		
	Vychytilova-Faltejskova		
	Khan		
	miRTarBase	*RASGRP1, BTG2, PDCD4*	
miR-155	Papaconstantinou		
	Frampton		
	miRTarBase	*CEBPB, TP53INP1, TAB2,*	
miR-181c	Chen	*MST1, LATS2, MOB1, SAV1*	Induces YAP/TAZ and expression of Hippo signaling genes
	miRTarBase	*ATP5G3, ZNF83, HSPA1B*	
miR-182	Li		
	miRTarBase	*FOX01, PTEN, CDKN1A*	Promotes cell proliferation and S phase of cell cycle
miR-196a	Bloomston		
	Kong		
	miRTarBase	*HOXB8, HOXC8, BACH1*	
miR-198	Vychytilova-Faltejskova		
	miRTarBase	*NNAT, MET, BIRC7, CCNT1*	
miR-203	Ikenaga		
	Greither		
	Frampton		
	miRTarBase	*SOCS3, SNAI1, ABL1, TP63*	
miR-222	Lee		
	Greither		
	Schultz		
	Frampton		
	miRTarBase	*CDKN1B, SOD2, PTEN*	
miR-301a	Xia	*SMAD4*	
	miRTarBase	*NKRF, MEOX2, RUNX3, PTEN*	
miR-371	He	*ING1*	Promotes cell proliferation
	miRTarBase	*PLEKHA1, DKK1, MYC*	
miR-486	Li		
	miRTarBase	*BCL11A, OGT, MKNK2*	
miR-744	Miyamae		Induces gemcitabine resistance
	miRTarBase	*CCL4, ppp1cab, MYC, EEF1A2*	
miR-1290	Li		Promotes cell proliferation and invasion
	miRTarBase	*CTC1, PER1, PRDX3, POP7*	

For each miRNA, we have included the most commonly cited proposed targets from the miRTarBase online database.

**Table 2:** List of down-regulated (potentially tumor suppressive) microRNAs in pancreatic cancer

miRNA	First author	Proposed target(s)	Proposed mechanism(s) and comments
miR-26	Deng	*Cyclin E2, EZH2*	G0/G1 cell cycle arrest
	miRTarBase	*CCND2, PTEN*	
miR-34a	Jamieson		
	Frampton		
	miRTarBase	*MYC, CDK6, E2F3, MDM4*	
miR-96	Li	*GPC1*	Inhibit proliferation, induce apoptosis, G0/G1 arrest
	miRTarBase	*FOX01, PFN1, KRAS*	
miR-130b	Zhao	*STAT3*	Inhibit proliferation/invasion, induce apoptosis, G0/G1 arrest
	miRTarBase	*BTBD3, ZNF800, RPS27A*	
miR-141	Zhu	*YAP1*	Inhibit proliferation, induce apoptosis
	miRTarBase	*ZEB2, ZEB1, YRDC*	
miR-142	Ohuchida		Effect only seen in setting of adjuvant gemcitabine
	miRTarBase	*TP53INP1, SIRT1, BTG3*	
miR-145	Gao	*Nanog (mRNA), ROR (lncRNA)*	Post-transcriptional silencing via binding to Nanog and ROR
	miRTarBase	*IRS1, FSCN1, POU5F1*	
miR-183	Zhou	*Bmi-1*	Suppress cell proliferation, regulate entry into S phase
	miRTarBase	*ANDEF1, EZR, ZNF138*	
miR-192	Botla	*Vimentin, Serpine-1*	Induce apoptosis, inhibit EMT/proliferation/invasion
	miRTarBAse	*SNRD, BCL2L11*	
miR-198	Marin-Muller	*PBX-1, VCP*	
	miRTarBase	*NNAT, MET, BIRC7, CCNT1*	Antagonize MSLN-mediated resistance to apoptosis
miR-200c	Yu	*E-cadherin*	Inhibits invasion, up-regulates proliferation (contradictory)
	miRTarBAse	*ZEB1, ZEB2, BMI1*	
miR-204	Ohuchida	*ING1*	Effect only seen in setting of adjuvant chemotherapy
	miRTarBase	*ZDHHC20, ZNF354B, BCL2*	
miR-218	Zhu		
	Li		
	miRTarBase	*LASP1, VOPP1, ROBO1*	
miR-323	Wang	*SMAD2, SMAD3*	Suppress TGF-b pathway, EMT, and cell motility
	miRTarBase	*SMAD2, SMAD3*	
miR-494	Ma		
	miRTarBase	*PTEN, CDK6, BMI1, ZEB1*	
miR-497	Xu	*FGF2, FGFR1*	Inhibit proliferation/migration/invasion, cell cycle arrest
	miRTarBase	*ANKEF1, AMOTL1*	
miR-506	Li	*SPHK1*	Induce apoptosis, inhibit proliferation, G0/G1 arrest
	miRTarBase	*QRFPR, CDK4, OLIG3*	
miR-545	Song	*RIG-1*	Inhibit cell growth
	miRTarBase		
miR-891b	Dong	*Cbl-b*	Promote p21 expression, inhibit cell proliferation
	miRTarBase	*CCND1, FZD6, CSDE1*	
miR-940	Song	*MyD88*	Inhibit cell growth
	miRTarBase	*AMOTL1, MYLIP, CDK4*	
miR-1181	Jiang	*SOX2, STAT3*	Inhibit cancer stem cell-like phenotypes
	miRTarBase	*NKX2-2, FOXP1*	

We have included the most commonly cited proposed targets from the online miRTarBase database.

**Table 3:** List of microRNA molecular signatures associated with overall survival

Publication	miRNA profiles associated with worse overall survival
Schultz et al.	*High*: 212, 675
	*Low*: let7g, 148a, 187
Greither et al.	*High*: 155, 203, 210, 222
Frampton et al.	*High*: 21, 23a, 27a
Namkung et al.	*High:* 574, 1244, 4474
	*Low:* 26b-star, 106b-star, 145-star, 324, 328, 564, 615, 668, 935, 943, 1292, 1914-star, 3194, 4321, 4746, 4763
Zhou et al.	*High:* 21, 193b, 583
	*Low*: 103-2, 125a, 126, 328, 340, 361, 374b, 454, 627, 664

### Studies reporting up-regulated (potentially oncogenic) micro-RNAs

#### miR-10b

Nakata et al. performed miRNA assays on 115 pancreatic cancer resection specimens and found that miR-10b was overexpressed in pancreatic cancer. They report that higher miR-10b levels are associated with increased tumor cell invasiveness and worse OS [14]. Similarly, Preis et al. performed fluorescence-based *in situ* hybridization (FISH) to characterize miRNA expression in 106 endoscopic ultrasound fine-needle aspiration (EUS-FNA) biopsies. They found that miR-10b was consistently overexpressed in PDAC. When expression levels were dichotomized, higher expression was associated with worse treatment response, shorter time to distant metastases, and worse OS [15].

#### miR-17

Yu et al. analyzed miR-17-5p expression levels in pancreatic cancer cell lines and formalin-fixed paraffin-embedded (FFPE) tissue samples from eighty surgical resection specimens. They found that pancreatic cancer demonstrates overexpression of miR-17-5p, and that higher miR-17-5p expression correlated with worse OS. Subsequent *in vitro* experiments suggested that miR-17-5p overexpression leads to higher cell growth ratios and promotes cancer cell invasion [16].

#### miR-21

Numerous publications have reported the prognostic implications of aberrantly overexpressed miR-21 in pancreatic cancer. Hu et al. conducted a systematic review of twelve publications discussing the prognostic role of miR-21. They conclude that elevated miR-21 expression levels significantly predict for worse OS in pancreatic cancer [17]. Khan et al. prospectively collected plasma samples from twelve patients with locally advanced unresectable pancreatic cancer. They used the plasma samples to quantify expression levels of circulating miR-21. When comparing patients with low versus high levels of circulating miR-21, they found that high miR-21 levels were associated with worse progression-free survival. There was also a trend toward worse overall survival [18]. Dillhoff et al. quantified miR-21 expression levels in eighty-five PDAC resection specimens. They detected miR-21 overexpression in PDAC relative to normal pancreatic tissue controls. In their cohort, high miR-21 levels were predictive of worse OS for patients with node-negative disease [19]. Kadera et al. also reported miR-21 overexpression in pancreatic tumor cells, as well as an association between high miR-21 levels and worse OS. Further *in vitro* analyses suggested that PDAC tumor cells induce tumor associated fibroblasts to upregulate miR-21 expression, thereby promoting tumor cell invasion and lymph node metastasis [20].

Nagao et al. performed miRNA expression profiling on sixty-five PDAC tissue specimens and found that miR-21 was the most consistently and significantly overexpressed miRNA (overexpressed in 75% of samples). High miR-21 levels correlated with worse OS in their cohort. Immunohistochemical (IHC) testing showed that miR-21 overexpression was associated with downregulated expression of programmed cell death 4 (PDCD4) and tissue inhibitor of metalloproteinase (TIMP3), both of which also correlated with worse OS [21].

Giovannetti et al. evaluated the prognostic implications of miR-21 by analyzing eighty-one PDAC patients treated with gemcitabine chemotherapy. Patients with higher miR-21 expression were found to have shorter OS. Functional *in vitro* studies suggested that miR-21 conferred chemoresistance via modulation of apoptosis, Akt phosphorylation, and expression of genes involved in cellular invasiveness [22]. Dhayat et al. quantified miRNA expression levels for ninety-eight patients with stage two pancreatic cancer treated with surgery and adjuvant gemcitabine. On multivariate analysis, overexpression of miR-21 and miR-100 were associated with worse progression-free survival (PFS) and OS [23]. Hwang et al. also suggest that miR-21 expression levels affect chemosensitivity by showing that low miR-21 expression correlated with longer OS in a cohort of patients who underwent surgery and adjuvant chemotherapy. There was no such correlation seen in patients who did not receive adjuvant chemotherapy. They validated their findings on a cohort of forty-five pancreatectomy specimens, all of which were treated with adjuvant therapy [24].

Jamieson et al. quantified miRNA expression in forty-eight prospectively collected pancreatic cancer tissue samples. On multivariate analysis, they report correlations between poor OS and high miR-21, low miR-30d, and low miR-34a. On a validation set of twenty-four patients, miR-21 and miR-34a expression levels remained significantly associated with OS [25].

In addition to tumor specimen sampling, there have been efforts to evaluate the prognostic utility of serum miR-21 levels. Liu et al. reported that elevated serum miR-21 levels are associated with worse OS based on univariate and multivariate analyses [26]. Wang et al. similarly took serum samples from 177 patients with unresectable or metastatic pancreatic cancer who received palliative gemcitabine-based chemotherapy. They found that serum miR-21 overexpression was an independent prognostic factor for both disease-free survival (DFS) and OS. *In vitro* analysis suggested that miR-21 confers chemoresistance by targeting Fas-ligand (FasL) and inhibiting gemcitabine-induced apoptosis [27].

Ma et al. conducted a comprehensive meta-review of published studies highlighting miRNA expression profiles in pancreatic cancer and found widespread agreement that miR-21 is overexpressed. In their own cohort of seventy patients with resectable disease, resection specimens with high miR-21, high miR-31, and low miR-375 were associated with worse OS on multivariate analysis [28].

#### miR-155

Papaconstantinou et al. investigated expression profiles of eight miRNA molecules (miR-21, miR-31, miR-122, miR-145, miR-146a, miR-155, miR-210, and miR-222) in 88 PDAC tissue samples and 98 control samples. They detected overexpression of miR-21 and miR-155 in cancer tissues. When clinical outcomes were analyzed, multivariate analysis found that high expression of miR-155 and miR-21 correlated with higher tumor stage and were independent factors in predicting worse overall survival [29].

#### miR-181c

Chen et al. published microarray analysis results for 136 pancreatic cancer samples and showed that miR-181c is significantly up-regulated in pancreatic cancer tissue. Statistical analysis showed that higher levels of miR-181c correlated with higher TNM stage and worse overall survival, compared to patients with low miR-181c expression. They also conducted *in vitro* studies which suggest that core components of the Hippo signaling pathway (*MST1*, *LATS2*, *MOB1*, and *SAV1*) may be targets of miR-181c. Western blotting analysis showed that miR-181c overexpression suppresses levels of Hippo signaling components and phosphorylation levels of downstream effectors, *YAP* and *TAZ*. In summary, overexpressed miR-181c appears to inactivate the tumor suppressive activity of the Hippo and YAP/TAZ signaling pathways, thereby leading to chemoresistance and worse overall survival [30].

#### miR-182

Li et al. conducted a study to determine relationships between miR-96-5p and miR-182-5p tumor expression and clinical outcomes in pancreatic cancer. They measured miR-182-5p expression in 38 tumor specimens and found that miR-182-5p is overexpressed in PDAC tissues. Higher miR-182-5p levels were associated with worse overall survival. *In vitro* studies showed that cells transfected with miR-182-5p mimics showed higher levels of proliferation and S-phase cell cycle arrest. Conversely, transfection with miR-182-5p inhibitors led to less proliferation and decreased time is S-phase [31].

#### miR-196a

A study by Bloomston et al. compared miRNA expression profiles of sixty-five PDAC tumor specimens versus forty-two chronic pancreatitis specimens. They identified twenty-five miRNAs that were differentially expressed in PDAC versus pancreatitis. When miRNA expression levels were dichotomized, they found that high expression of miR-196a was associated with shorter OS [32].

Kong et al. quantified miR-196a expression in serum samples from thirty-five PDAC patients. They found that miR-196a expression was significantly higher in patients with unresectable disease (stage III–IV) versus those with resectable disease (stage I–II). Additionally, median OS was significantly shorter for patients with high serum levels of miR-196a, making this a potential non-invasive prognostic marker [33].

#### miR-203

Ikenaga et al. quantified miRNA expression in 113 PDAC tissue samples, twenty pancreatitis samples, and eight normal pancreatic tissue samples. They found that miR-203 was overexpressed in PDAC. On univariate and multivariate analyses, high expression of miR-203 was an independent predictor of worse OS [34]. Additionally, a paper by Greither et al. includes miR-203 in a panel of four miRNAs (miR-155, miR-203, miR-210, miR-222) synergistically associated with overall survival [35].

#### miR-222

MiR-222 has been shown to play a role in cancer cell proliferation. Lee et al. prospectively collected sixty matched pairs of PDAC tissues and adjacent normal pancreatic tissues. They quantified miR-222 expression in each sample and found that miR-222 was significantly elevated in PDAC. Additionally, miR-222 expression directly correlated with Ki67 expression and predicted for worse overall survival [36].

#### miR-301a

Xia et al. utilized tissue microarray analysis to quantify miR-301a expression in ninety PDAC tumor specimens after surgery. MiR-301a was overexpressed in PDAC samples, and even more so in patients with more advanced disease. Higher expression of miR-301a was an independent predictor of higher nodal staging and worse OS. Functional *in vitro* and *in vivo* studies suggested that miR-301a acts as an oncogene by directly targeting and repressing SMAD4 [37].

#### miR-371

He et al. investigated the roles of miR-371-5p in PDAC by quantifying expression levels in seventy-five tumor specimens and fifteen normal tissue control specimens. They found that miR-371-5p was significantly upregulated in PDAC. High miR-371-5p expression was significantly associated with shorter OS. *In vitro* and *in vivo* assays demonstrated that miR-371 overexpression is associated with downregulated inhibitor of growth 1 (ING1) and increased cell proliferation. They concluded that aberrantly overexpressed miR-371 functions as an oncogene within an ING1/miR-371 regulatory feedback loop [38].

#### miR-486 and miR-1290

Li et al. utilized TaqMan MicroRNA Arrays to measure pre-operative serum expression levels of 735 miRNAs in 81 patients with resectable PDAC. They compared expression levels to those of healthy controls and sought to determine if any miRNAs were prognostic. They found that miR-1290 was the most diagnostic miRNA with a receiver operating characteristic (ROC) analysis showing an area under the curve (AUC) of 0.96. Serum miR-1290 was overexpressed in PDAC patients. When dichotomized about median expression levels, high miR-1290 and miR-486-3p expression predicted worse overall survival after surgery. *In vitro* testing suggested that miR-1290 influenced cell proliferation and invasive ability. They conclude that miR-1290 has diagnostic and prognostic utility in resectable pancreatic cancer [39].

#### miR-744

Miyamae et al. conducted a study to explore novel plasma miRNAs for screening and prognosis in pancreatic cancer. They analyzed serum samples of ninety-four PDAC patients and sixty-eight healthy volunteers. They found elevated expression levels of miR-744 in PDAC patients. When comparing pre-operative versus postoperative serum miR-744 levels, they found a significant reduction after surgery. High expression of miR-744 was an independent prognostic factor for worse OS after surgery. It was also prognostic in patients with inoperable disease treated with chemotherapy. *In vitro* studies supported this result by showing that overexpression of miR-744 induced significant chemoresistance to gemcitabine [40].

### Studies reporting down-regulated (potentially tumor suppressive) micro-RNAs

#### miR-26a

A study by Deng et al. examined the role of miR-26a in pancreatic tissue by quantifying its expression levels in 106 PDAC tissue samples. They found that miR-26a expression was downregulated in PDAC compared to adjacent normal tissue. Low expression of miR-26a was associated with shorter OS. Further *in vitro* assays showed that miR-26a overexpression was associated with cyclin E2 downregulation, cell cycle arrest, inhibition of cell proliferation, and decreased tumor growth [41].

#### miR-34a

There are two publications that have correlated miR-34a expression levels to overall survival in pancreatic cancer. First, Jamieson et al. quantified miRNA expression profiles of 48 PDAC tumor samples and found that low expression of miR-34a was associated with worse overall survival on multivariate analysis. A meta-analysis of miRNAs aberrantly expressed in pancreatic cancer was performed by Frampton et al [42]. They identified 5 significantly up-regulated miRNAs, and 1 down-regulated miRNA. In particular, they identified low tumor levels of miR-34a as predictive of worse overall survival. Ji et al. studied the effects of transfecting miR-34a mimics in p53-mutant cell lines and detected suppression of Bcl-2 and Notch1/2. Further *in vitro* and *in vivo* studies found that miR-34 restoration inhibits clonogenic cell growth/invasion, promotes cell cycle arrest, and induces apoptosis. Their results suggest that, in p53-deficient pancreatic cancer, miR-34a restores the tumor suppressive function of p53 via regulation of Bcl-2 and Notch1/2 [43].

#### miR-96

In addition to reporting the effects of miR-182-5p, the previously sited publication by Li et al. also studied the effects of miR-96-5p in 38 pancreatic cancer tissue specimens. They report that miR-96-5p is downregulated in PDAC and lower expression levels were associated with worse overall survival. *In vitro* functional studies showed that cells transfected with miR-96-5p mimics exhibited lower proliferation rates, more G0/G1 cell cycle arrest, and more apoptosis. Dual luciferase assays showed that miR-96-5p directly regulates Glypican 1 (*GPC1*).

#### miR-130b

A study by Zhao et al. reported that miR-130b is downregulated in PDAC. On univariate analysis, low miR-130b expression correlated with higher TNM stage, distant metastases, and worse prognosis. On multivariate analysis, miR-130b expression was an independent prognostic factor. *In vitro* and *in vivo* functional studies indicated that miR-130b acts as a tumor suppressor by targeting STAT3, inducing apoptosis, and promoting cell cycle arrest [44].

#### miR-141

A study by Zhu et al. explored the expression levels and mechanisms of action of miR-141. They evaluated ninety-four tissue samples and found that miR-141 is downregulated in PDAC compared to adjacent normal tissue. Low expression was associated with more advanced stage and shorter OS. Functional studies suggested that miR-141 inhibits tumor growth by targeting Yes-associated protein-1 (YAP1) and enhancing caspase-3-dependent apoptosis [45].

#### miR-142 and miR-204

Ohuchida et al. obtained macro-dissected FFPE resection specimens, extracted total RNA, and quantified miRNA expression profiles for 90 PDAC patients treated with or without gemcitabine after surgical resection. They found that, in the cohort of patients receiving adjuvant gemcitabine chemotherapy, those with high miR-142-5p and miR-204 expression had better overall survival. However, this prognostic finding was not seen in the group not receiving gemcitabine. Their results suggest that high miR-142-5p and miR-204 predict for chemo-sensitivity and improved OS in patients treated with gemcitabine [46].

#### miR-145

Gao et al. conducted *in vitro* and *in vivo* studies to characterize the roles of ROR (a long non-coding RNA molecule) in regulating the pathogenesis and progression of pancreatic cancer. They also performed bioinformatics analysis and luciferase assays to determine the interactions between ROR and Nanog (mRNA) with miR-145. They found that ROR is up-regulated in pancreatic tumor cells and associated with worse overall survival. They also report an inverse relationship between ROR expression and miR-145 expression, suggesting that low miR-145 expression is associated with worse overall survival. Their bioinformatics analysis showed that there are common binding sites for miR-145 found on both ROR and Nanog. They propose that miR-145 can induce post-transcriptional silencing of its target genes by binding to specific sites on ROR or Nanog [47].

#### miR-183

A study by Zhou et al. sought to examine the role of miR-183 in pancreatic cancer. They report that miR-183 was downregulated in PDAC tissue samples. Low miR-183 expression was significantly associated with higher histologic grade, more advanced TNM stage, and shorter OS. Functional studies revealed an inverse correlation between expression of miR-183 and B-cell-specific moloney murine leukemia virus insertion site 1 (Bmi-1), with downstream effects seen on levels of cyclin D1, CDK2, and CDK4. They conclude that miR-183 exerts tumor suppressor effects by targeting Bmi-1, but low expression in pancreatic cancer contributes to worse prognosis [48].

#### miR-192

Botla et al. studied miR-192 expression levels in 94 pancreatic cancer tissue samples. They found that miR-192 is epigenetically downregulated in PDAC, via promoter methylation. Additionally, they found an association between high miR-192 expression and better overall survival. *In vitro* analysis showed an inverse correlation between miR-192 expression and E-cadherin and vimentin, both epithelial-mesenchymal transition markers. They also report that miR-192 appears to directly regulate *SERPINE1* protein expression. Functionally, overexpression of miR-192 was shown to reduce PDAC tumor cell proliferation, induce apoptosis, and regulate invasion [49].

#### miR-198

Marin-Muller et al. investigated the regulation of mesothelin by aberrantly expressed miR-198 in pancreatic cancer. They found that miR-198 is downregulated in PDAC and low levels are associated with worse OS. Based on *in vivo* studies, they propose that miR-198 is repressed by mesothelin via NF-ĸB mediated OCT-2 induction. Repression of miR-198 leads to overexpression of mesothelin, pre-B-cell leukemia homeobox factor 1 (PBX-1) and valosin-containing protein (VCP). These downstream targets are, in turn, associated with increased tumor proliferation and invasion, suggesting that miR-198 acts as a tumor suppressor that is under-expressed in PDAC [50].

There is opposing evidence for the role of miR-198, however. Another study by Vychytilova-Faltejskova et al. reported that miR-198 was overexpressed in PDAC tumor tissues compared to healthy pancreatic tissue. Higher expression of miR-198 was associated with worse disease-free and overall survival in their cohort [51]. This was the only instance of discordant findings between studies with regard to miRNA up- or down-regulation in pancreatic tumor specimens.

#### miR-200c

The miR-200 family has been reported to function as tumor suppressors by regulating epithelial-to-mesenchymal transition (EMT). Specifically, miR-200c has been associated with upregulating E-cadherin expression, a key gene involved in regulating EMT. Yu et al. evaluated miR-200 in ninety-nine PDAC tissue samples and found that high levels of miR-200c were associated with improved OS rates. They also confirmed a direct correlation between miR-200c and E-cadherin levels, suggesting that they function together as tumor suppressors [52].

#### miR-218

Zhu et al. examined the prognostic value of miR-218. Using qRT-PCR, they quantified miR-218 expression in 113 PDAC tissue samples and thirty-three normal pancreatic tissue samples. They found that miR-218 is significantly downregulated in PDAC. Univariate analyses showed that low miR-218 expression was associated with higher histologic grade, more advanced tumor stage, higher rates of recurrence, and worse PFS. On multivariate analysis, low levels of miR-218 were associated with shorter OS [53]. A similar study was published by Li et al., who reported concordant findings that miR-218 is under-expressed in PDAC. In this study, low miR-218 levels were associated with higher stage, higher histologic grade, distant metastases, and worse OS [54].

#### miR-323

Wang et al. studied miR-323-3p expression levels in 108 PDAC tumor specimens. They found that miR-323-3p is significantly downregulated in PDAC tissue and low expression levels were associated with shorter overall survival and metastasis-free survival. *In vitro* and *in vivo* studies suggested that miR-323-3p upregulates E-cadherin and α-catenin, while also downregulating vimentin and N-cadherin. Functionally, miR-323-3p was associated with inhibited cell motility and metastatic potential. Further Western blotting and luciferase reporter assays revealed that SMAD2 and SMAD3 protein expression was markedly decreased in miR-323-3p transduced PDAC cells, whereas inhibition of miR-323-3p had the opposite effect. SMAD2 and SMAD3 phosphorylation and translocation to the nucleus are critical steps in TGF-β signal transduction. They demonstrated that SMAD2 and SMAD3 phosphorylation and nuclear accumulation were reduced and TGF-β activity was suppressed in PDAC cells following overexpression of miR-323-3p. In summary, they conclude that downregulation of miR-323-3p in PDAC tissue leads to increased expression of SMAD2 and SMAD3, which contributes to more aggressive tumor phenotypes and worse overall survival [55].

#### miR-494

Ma et al. extracted total RNA from 99 PDAC resection tissues and matched normal adjacent pancreatic tissue to evaluate the clinical significance of miR-494. They found that miR-494 is significantly down-regulated in PDAC. Low levels of miR-494 were associated with higher TNM stage, higher histologic grade, more lymphovascular invasion, and more frequent distant metastases. Additionally, patients with low expression had shorter overall survival, suggesting that reduced miR-494 is an independent, poor prognostic factor [56].

#### miR-497

Xu et al. report that miR-497 expression is significantly downregulated in PDAC compared with tumor-adjacent samples. Low miR-497 expression was an independent predictor of shorter OS. Functional studies suggested that miR-497 acts as a tumor suppressor by regulating cell proliferation, migration, and invasion. Additionally, they conclude that miR-497 is a chemo-sensitizer by decreasing the percentage of cells in the relatively resistant S phase. *In vitro* studies showed that fibroblast growth factor 2 and fibroblast growth factor 1 are direct targets of miR-497 [57].

#### miR-506

Sequencing analyses by Li et al. revealed that the miR-506 promoter is highly methylated and under-expressed in pancreatic cancer compared to normal tissue controls. They report that low miR-506 expression is significantly associated with higher clinical stage, higher pathologic stage, higher distant metastasis rates, and decreased OS. Functional studies suggested that miR-506 acts as a tumor suppressor by inhibiting cell proliferation, enhancing apoptosis, and inducing G1/S cell cycle arrest. *In vitro* experiments identified sphingosine kinase 1 (SPHK1) as a direct target of miR-506. Low miR-506 expression, therefore, leads to high SPHK1 expression which is also associated with decreased survival in PDAC [58].

#### miR-545

Song et al. investigated the role of miR-545 in PDAC by performing qRT-PCR on seventy-eight surgical resection specimens. They found that miR-545 is under-expressed in tumor tissue and low expression was associated with decreased OS. They also identified an inverse relationship between expression levels of miR-545 and retinoid-inducible gene 1 (RIG-1). Based on functional studies, they conclude that low miR-545 expression in PDAC promotes tumor cell growth and reduced OS via up-regulation of RIG-1 [59].

#### miR-891b

Dong et al. performed microRNA microarray analysis to compare expression profiles of good versus poor prognosis groups (median overall survival 48.0 versus 6.3 months). They report that miR-891b was significantly up-regulated in the good prognostic group. When the cohort was dichotomized based on the median miR-891b expression level, high miR-891b expression was significantly associated with better overall survival. They validated these findings on an independent cohort of 114 pancreatic cancer samples, again showing a strong correlation between high levels of miR-891b and improved overall survival based on univariate and multivariate analyses. They then conducted *in vitro* studies which showed that up-regulation of miR-891b leads to suppressed tumor cell growth, a finding that was confirmed by an *in vivo* xenograft mouse model. Further testing showed that elevated miR-891b was associated with suppressed Cbl-b protein expression via post-transcriptional silencing. Cbl-b protein silencing was found to suppress tumor cell growth and correlate with lower Ki-67 values. In summary, this paper reports that miR-891b is a prognostic marker with tumor suppressive functions mediated by post-transcriptional silencing of Cbl-b gene products [60].

#### miR-940

Song et al. tested the levels of miR-940 in seventy-eight PDAC tissue samples and cell lines. They also performed *in vitro* studies by manipulating miR-940 expression levels via miRNA mimic transfection and antisense oligonucleotides transfection. They report that low miR-940 expression correlated with worse OS. The functional studies revealed that low miR-940 is associated with higher rates of cell growth. They utilized a bioinformatics algorithm and a dual luciferase reporter assay to identify myeloid differentiation primary response gene – 88 (MyD88) as miR-940's primary target [61].

#### miR-1181

Jiang et al. quantified miR-1181 expression levels in eighty paired human pancreatic cancer tissues with matched adjacent normal tissues. They report miR-1181 downregulation in PDAC tissue samples. They also found associations between low miR-1181 expression, shorter time to distant metastases, and shorter DFS and OS. Based on functional *in vitro* experiments, they found that miR-1181 functions as a tumor suppressor by inhibiting cancer stem cell-like phenotypes. They demonstrated that miR-1181 directly suppresses sex-determining region Y-box 2 (SOX2) and signal transduction and activation of transcription 3 (STAT3) expression, resulting in inhibition of the STAT3 pathway [62].

### Studies reporting on the development of prognostic micro-RNA signatures

#### miR-212, miR-675, miR-148a, miR-187, miR-let-7g

Schultz et al. evaluated 225 surgical specimens in an effort to identify a miRNA expression prognostic index that can predict overall survival after radical surgery. They utilized the Lasso (Least Absolute Shrinkage and Selection Operator) statistical method in conjunction with Cox proportional hazard modeling to relate miRNA expression to OS. They found that a prognostic index (PI) consisting of high miR-212, high miR-675, low miR-148a, low miR-187, and low miR-let-7g predicted shorter OS. When the prognostic index scores were dichotomized about the median, median survival was 1.09 years for PI greater than median versus 2.23 years for PI less than median [63].

#### miR-155, miR-203, miR-210, miR-217, miR-222

A study by Greither et al. focused on measuring expression levels of six miRNAs which had previously been shown to be differentially expressed in pancreatic tumors. By utilizing multivariate Cox's regression hazard analyses, they found a significant correlation between expression levels of four individual miRNAs (high miR-155, high miR-203, high miR-210, and high miR-222) and shorter OS. Patients who had elevation of all 4 miRNAs had a 6.2-fold increased risk of death related to pancreatic cancer, compared to patients with low expression. Combining the 4 miRNAs gave the best predictive power, as patients with elevated expression of all four micro-RNAs had approximately three-fold higher risk of dying than the effect of any individual miRNA [35].

#### miR-21, miR-23a, miR-27a

Frampton et al. combined miRNA and mRNA expression profile data for ninety-one patients and reported that high levels of miR-21, miR-23a, and miR-27a were associated with shorter OS after pancreatectomy. Based on *in vitro* data, they propose that those three miRNAs act synergistically in repressing a network of tumor suppressors including PDCD4 [21], BTG2, and NEDD4L. [64] In an effort to form more firm conclusions regarding the prognostic impact of miRNA expression profiling in pancreatic cancer, the same group conducted a meta-analysis which concluded that OS is significantly shortened in patients with high tumor levels of miR-21. Additionally, they report that high miR-155, high miR-203, high miR-222, high miR-10b, and low miR-34a are all associated with worse OS.

#### miR-21, miR-103-2, miR-125a, miR-126, miR-193b, miR-328, miR-340, miR-361, miR-374b, miR-454, miR-584, miR-627, miR-664

Zhou et al. sought to identify a microRNA signature panel that could predict prognosis of pancreatic cancer based on overall survival data from The Cancer Genome Atlas. Based on a cohort of 167 patients, a Cox proportional regression model was used to identify microRNAs significantly associated with overall survival. A total of thirteen microRNAs (Table 3) were identified to be significantly related with overall survival. There were ten tumor suppressor miRNAs identified: miR-103-2, miR-125a, miR-126, miR-328, miR-340, miR-361, miR-374b, miR-454, miR-627 and miR-664 and three oncogenic miRNAs identified: miR-21, miR-193b, and miR-584. Based on these results, a risk score formula was developed to create high and low risk prognostic groups, dichotomized by median risk score. Univariate and multivariate analyses showed a statistically significant relationship between the microRNA risk score and overall survival [65].

#### Prognostic subtypes based on miRNA expression profiles

Namkung et al. conducted an interesting study to identify prognosis-related molecular subtypes of PDAC using miRNA expression profiling. They performed microarray analysis of expression levels of 1733 unique miRNAs in 104 pancreatic tumor samples. They applied statistical models to detect patient subgroups with miRNA expression profiles that were informative in predicting OS. They identified three PDAC tumor subtypes, two of which had similar overall prognosis and were grouped together, based on expression profiles of nineteen miRNA molecules. On multivariate analysis, the molecular subgroups were independent predictors of overall survival. The “high risk” subgroup was associated with up-regulated miR-574-5p, miR-1244, miR-4474-5p and down-regulated miR-106b-star, miR-324-3p, miR-943, miR-1292, miR-935, miR-668, miR-4763-5p, miR-145-star, miR-328, miR-26b-star, miR-615-5p, miR-1914-star, miR-564, miR-3194-5p, miR-4746-3p, and miR-4321. The median DFS of the high and low risk groups were 6.5 and 17.3 months, respectively (*p* = 0.01). Median OS times were 17.2 and 30.5 months for the high risk group and low risk group, respectively (*p* = 0.009). When each of the nineteen miRNAs from the risk group was analyzed for its association with prognosis, eleven of them were independently associated with worse OS (high miR-574-5p and miR-1244; low miR-342-3p, miR-943, miR-145-star, miR-328, miR-26b-star, miR-1914-star, miR-564, miR-3194-5p, and miR-4321) [66].

### Summary of miRNAs dysregulated in pancreatic cancer and their potential contributions to various cellular process and signaling pathways

**Figure 1:**
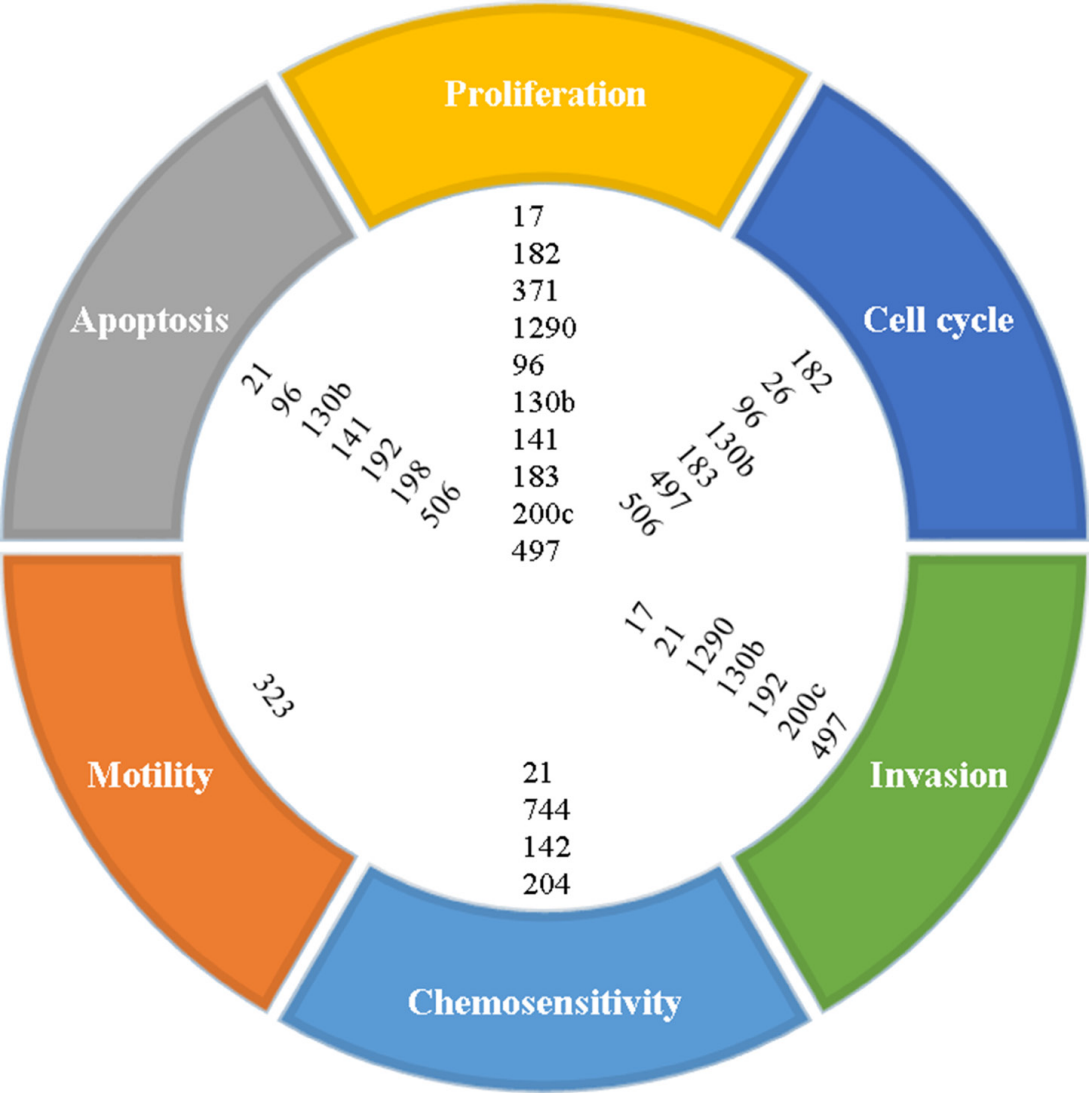
Proposed cellular processes affected by microRNAs based on reported functional studies

**Figure 2:**
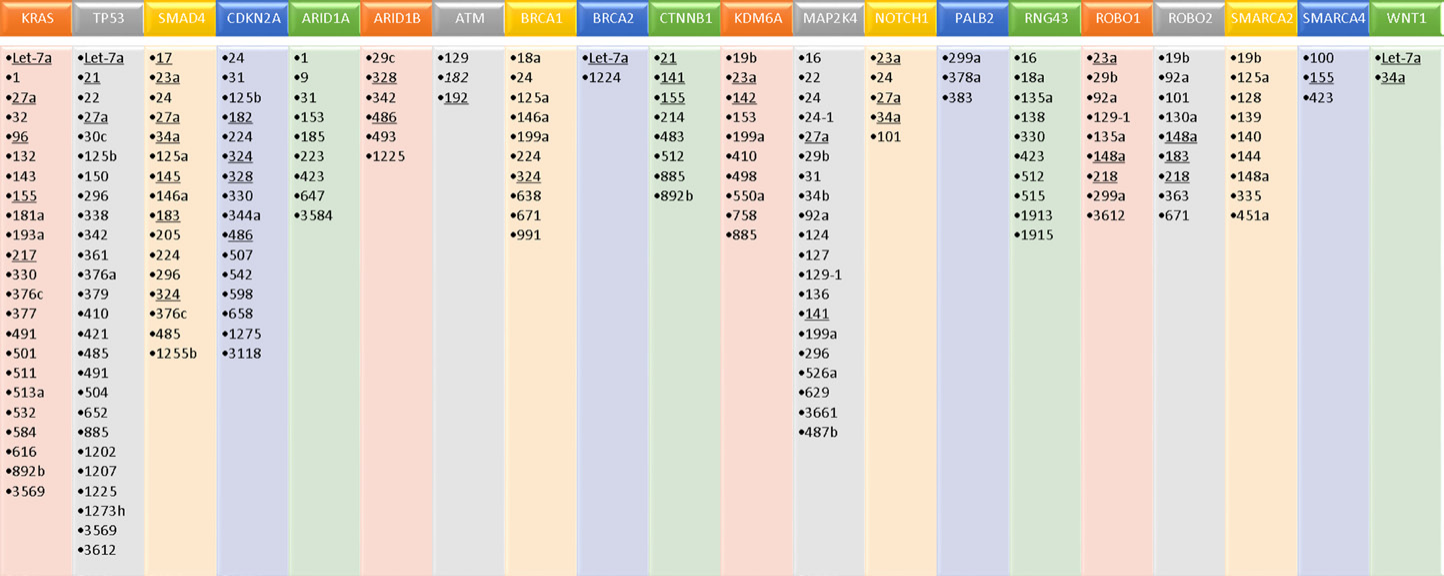
MicroRNAs identified as being associated with significantly mutated genes in pancreatic cancer, based on ingenuity pathway analysis (IPA) MiRNAs that were also identified as dysregulated in pancreatic cancer, based on our literature review, are underlined.

The hallmarks of cancer include such processes as tumor cell proliferation, dysregulation of the cell cycle, increased motility, increased invasion, resistance to apoptosis, and resistance to chemotherapy. Many of the miRNAs reported in this paper are implicated in altering or promoting these processes, as shown in Figure 1. In addition, a number of elegant genomic studies using deep sequencing and RNA expression methodologies have carefully delineated the genomic aberrations and aberrant signaling pathways that may be driving pancreatic cancer [67–69]. Significantly mutated genes in pancreatic cancer identified in these studies include KRAS, TP53, SMAD4/DPC4, CDKN2A, ARID1A, ARID1B, KDM6A, WNT, CTNNB1, NOTCH1, SMARCA4, SMARCA2, BRCA1, BRCA2, ATM, PALB2, MAP2K4, RNF43, ROBO1, and ROBO2. Thus, the aberrant cellular pathways that are hallmarks of pancreatic cancer include RAS-MAPK, WNT/NOTCH, DNA damage control, DNA repair, cell cycle maintenance, TGFβ signaling, chromatin modification and SWI/SNF pathway, and the ROBO/SLIT pathway. Using Ingenuity Pathway Analysis (IPA), we have identified miRNAs implicated in the signaling and/or function of some of these genes. As shown in Figure 2, many of the miRNAs identified in this review have direct or indirect relationships to these altered genes.

## DISCUSSION

The growing body of literature on miRNA expression profiling and mechanisms of action in pancreatic cancer suggests that these molecules play important roles in this disease. Aberrant expression of specific miRNAs with oncogenic and/or tumor suppressor properties likely triggers cellular signaling cascades involved in malignant transformation, proliferation, invasion, and metastasis [70]. There is also data to suggest that miRNA expression levels correlate with treatment response by rendering tumors more or less chemo-sensitive. The data presented here represents our current level of evidence for the prognostic value of miRNA expression profiling in predicting clinical outcomes for pancreatic cancer. Clearly, there are numerous individual microRNAs that have been correlated with prognosis. Unfortunately, only a few miRNAs have multiple studies validating their prognostic capability. Whether this is a reflection of publication bias (i.e. the tendency to only report novel scientific findings), use of different technologies that enrich for certain miRNAs, different patient populations, or that some of the miRNAs identified were discovered as statistical “false positives” (type 1 error) is not clear.

As reviewed here, several miRNAs have multiple publications validating their prognostic utility, including miR-10b, miR-21, miR-34a, miR-155, miR-196a, miR-198, miR-200c, miR-203, miR-210, miR-218, miR-222, and miR-328. The vast majority of these studies confirm that a particular miRNA is associated with the same trend in expression (up-regulated or down-regulated in PDAC) and clinical outcome, although there is one exception (miR-198). By far, the most reported miRNA was miR-21. Numerous publications report consistent results that miR-21 is oncogenic and aberrantly overexpressed in pancreatic cancer. When cohorts are dichotomized according to miR-21 expression levels, results have consistently shown that high miR-21 expression is associated with worse overall survival. *In vitro* studies by Moriyama et al. suggest that elevated miR-21 increases cancer cell proliferation, invasion, and gemcitabine resistance by targeting matrix metalloproteinase-2, metalloproteinase-9, and VEGF [71]. Alternatively, it has been proposed that miR-21 downregulates expression of programmed cell death 4 (PDCD4) and tissue inhibitor of metalloproteinase (TIMP3), both of which function as tumor suppressors [21]. Some studies report that miR-21 overexpression only correlates with overall survival in patients receiving post-operative chemotherapy, suggesting that tumor intrinsic miR-21 may promote cellular resistance to chemotherapy and that miR-21 may be a predictive biomarker of chemotherapy response.

This review highlights the current state of the field on aberrant miRNA expression and the ability of miRNAs to provide prognostic information for patients with pancreatic cancer. In addition, there is clear evidence that miRNAs are linked to a number of cellular functions that promote tumor progression, including proliferation, invasion/migration, apoptosis resistance, dysregulated cell cycle maintenance, and resistance to therapy (Figure 1). Furthermore, a number of miRNAs are linked to pathways that have been identified to be significantly altered in pancreatic cancer, including RAS-MAPK, Wnt-Notch signaling, SWI/SNF chromatin modifying pathways, TGFβ signaling, cell cycle dysregulation, DNA damage response and repair, and ROBO/SLIT signaling (Figure 2). More preclinical studies are needed to better define the relationship of the dysregulated miRNAs identified in this review on oncogenic and/or tumor suppressive properties in pancreatic cancer cells, as well as the tumor microenvironment. A better understanding of the mechanisms by which these miRNAs promote various cancer cell functions such as survival, invasion, metastasis, resistance to therapy, etc. could lead to the discovery of novel therapeutic targets.

With regards to the prognostic ability of miRNAs, further studies are needed to validate these miRNAs for their prognostic (and predictive) ability, both retrospectively and prospectively. This information could create opportunities to identify new targeted therapies that counteract the pathologic cell signaling of aberrantly expressed miRNAs. In order to maximize the prognostic impact and clinical utility of miRNA expression profiling, efforts should be focused on developing panels of the most significant miRNAs, similar to the studies by Schultz, Greither, and Namkung et al [35, 63, 66]. While single miRNAs offer opportunities to understand the underlying biology and develop new therapeutic strategies, it is unlikely that expression of a single miRNA can reliably predict a particular clinical outcome with high sensitivity and specificity, given the heterogeneity of expression across different patients. Rather, risk profiles generated by integrating expression of multiple miRNAs are more likely to provide substantial and reproducible predictive power for detecting disease, predicting patterns of failure, and determining overall prognosis. Together with clinical prognostic factors, such molecular tools have the potential to assist clinicians with patient selection for various treatment modalities including surgery, systemic therapy, and radiotherapy.

In summary, the studies identified in this review suggest that miRNAs have the ability to prognosticate in pancreatic cancer based on tissue or readily-available serum. In this review, we have presented data on 72 individual miRNAs (23 up-regulated, 49 down-regulated) that have been demonstrated to correlate with survival in pancreatic cancer. Aside from miR-21, there are few individual miRNAs that have extensive validation, perhaps due to different patient populations, treatment strategies, methodologies, and technologies used in these studies. Indeed, to our knowledge, none of these miRNAs have been implemented as prognostic markers in routine practice. It is likely that molecular signatures incorporating multiple miRNAs will have improved prognostic ability compared to individual miRNAs. Standardization of technology and methodology, as well as validation across multiple datasets, will provide the best opportunity for miRNA profiling to be integrated into clinical practice. Finally, a better understanding of mechanisms by which miRNA dysregulation promotes aberrant cellular and molecular signaling in tumor cells will allow the identification of novel molecular targets.

## Abbreviations

PDAC: pancreatic ductal adenocarcinoma
miR: microRNA
miRNA: microRNA
mRNA: messenger RNA
DNA: deoxyribonucleic acid
RNA: ribonucleic acid
OS: overall survival
LRR: locoregional recurrence
CRT: chemoradiotherapy
ASCO: American Society of Clinical Oncology
FISH: fluorescence-based *in situ* hybridization
FFPE: formalin-fixed paraffin embedded
IHC: immunohistochemical
